# The population genomic legacy of the second plague pandemic

**DOI:** 10.1016/j.cub.2022.09.023

**Published:** 2022-11-07

**Authors:** Shyam Gopalakrishnan, S. Sunna Ebenesersdóttir, Inge K.C. Lundstrøm, Gordon Turner-Walker, Kristjan H.S. Moore, Pierre Luisi, Ashot Margaryan, Michael D. Martin, Martin Rene Ellegaard, Ólafur þ. Magnússon, Ásgeir Sigurðsson, Steinunn Snorradóttir, Droplaug N. Magnúsdóttir, Jason E. Laffoon, Lucy van Dorp, Xiaodong Liu, Ida Moltke, María C. Ávila-Arcos, Joshua G. Schraiber, Simon Rasmussen, David Juan, Pere Gelabert, Toni de-Dios, Anna K. Fotakis, Miren Iraeta-Orbegozo, Åshild J. Vågene, Sean Dexter Denham, Axel Christophersen, Hans K. Stenøien, Filipe G. Vieira, Shanlin Liu, Torsten Günther, Toomas Kivisild, Ole Georg Moseng, Birgitte Skar, Christina Cheung, Marcela Sandoval-Velasco, Nathan Wales, Hannes Schroeder, Paula F. Campos, Valdís B. Guðmundsdóttir, Thomas Sicheritz-Ponten, Bent Petersen, Jostein Halgunset, Edmund Gilbert, Gianpiero L. Cavalleri, Eivind Hovig, Ingrid Kockum, Tomas Olsson, Lars Alfredsson, Thomas F. Hansen, Thomas Werge, Eske Willerslev, Francois Balloux, Tomas Marques-Bonet, Carles Lalueza-Fox, Rasmus Nielsen, Kári Stefánsson, Agnar Helgason, M. Thomas P. Gilbert

**Affiliations:** 1 The GLOBE Institute, Faculty of Health and Medical Sciences, University of Copenhagen, Øster Farimagsgade 5A, 1353 Copenhagen, Denmark; 2 deCODE Genetics, AMGEN Inc., Sturlugata 8, 102 Reykjavík, Iceland; 3 Department of Anthropology, School of Social Sciences, University of Iceland, Gimli, Sæmundargata, 102 Reykjavík, Iceland; 4 National Yunlin University of Science & Technology, 123 University Road, Section 3, 64002 Douliu, Yun-Lin County, Taiwan; 5 Department of Archaeology and Anthropology, National Museum of Natural Science, 1 Guanqian Road, North District Taichung City 404023, Taiwan; 6 Facultad de Filosofía y Humanidades, Universidad Nacional de Córdoba, Córdoba, Argentina; 7 Microbial Paleogenomics Unit, Institut Pasteur, 25-28 Rue du Dr Roux, 75015 Paris, France; 8 NTNU University Museum, Norwegian University of Science and Technology (NTNU), 7491 Trondheim, Norway; 9 Department of Archaeological Sciences, Faculty of Archaeology, Leiden University, Leiden, the Netherlands; 10 UCL Genetics Institute, Department of Genetics, Evolution and Environment, University College London, Darwin Building, Gower Street, London WC1E 6BT, UK; 11 Department of Biology, University of Copenhagen, Ole Maaløes Vej 5, 2200 Copenhagen, Denmark; 12 International Laboratory for Human Genome Research, Laboratorio Internacional de Investigación sobre el Genoma Humano (LIIGH), Universidad Nacional Autónoma de México (UNAM), 3001 Boulevard Juriquilla, 76230 Querétaro, Mexico; 13 Illumina Artificial Intelligence Laboratory, Illumina Inc., San Diego, CA, USA; 14 Novo Nordisk Foundation Center for Protein Research, University of Copenhagen, Blegdamsvej 3, 2200 Copenhagen, Denmark; 15 Institute of Evolutionary Biology (UPF-CSIC), PRBB, Dr. Aiguader 88, 08003 Barcelona, Spain; 16 Catalan Institution of Research and Advanced Studies (ICREA), Passeig de Lluís Companys, 23, 08010 Barcelona, Spain; 17 CNAG-CRG, Centre for Genomic Regulation (CRG), Barcelona Institute of Science and Technology (BIST), Baldiri i Reixac 4, 08028 Barcelona, Spain; 18 Institut Català de Paleontologia Miquel Crusafont, Universitat Autònoma de Barcelona, Edifici ICTA-ICP, c/ Columnes s/n, 08193 Cerdanyola del Vallès, Barcelona, Spain; 19 Museu de Ciències Naturals de Barcelona, 08019 Barcelona, Spain; 20 Department of Evolutionary Anthropology, University of Vienna, Vienna, Austria; 21 Max Planck Institute for the Science of Human History, Kahlaische Strasse 10, 07745 Jena, Germany; 22 Institute for Archaeological Sciences, University of Tübingen, Tübingen, Germany; 23 Museum of Archaeology, University of Stavanger, Stavanger, Norway; 24 China National GeneBank, BGI-Shenzhen, Shenzhen 518083, China; 25 Evolutionsbiologisk Centrum EBC, Norbyv. 18A, 752 36 Uppsala, Sweden; 26 KU Leuven, Herestraat 49, 3000 Leuven, Belgium; 27 Institute of Genomics, University of Tartu, Riia 23b, 51010 Tartu, Estonia; 28 Department of Business, History and Social Sciences, University of South-Eastern Norway, Notodden, Norway; 29 EA – Eco-anthropologie (UMR 7206), Muséum National d’Histoire Naturelle, CNRS, Université Paris Diderot, Paris, France; 30 Department of Archaeology, Kings Manor and Principals House, University of York, Exhibition Square, York YO1 7EP, UK; 31 CIIMAR, Centro Interdisciplinar de Investigação Marinha e Ambiental, Universidade do Porto, Terminal de Cruzeiros do Porto de Leixões, Avenida General Norton de Matos, Matosinhos, Portugal; 32 Centre of Excellence for Omics-Driven Computational Biodiscovery (COMBio), Faculty of Applied Sciences, Asian Institute of Medicine, Science and Technology (AIMST), 08100 Bedong, Kedah, Malaysia; 33 Biobank1, St. Olavs Hospital HF, Trondheim, Norway; 34 School of Pharmacy and Biomolecular Sciences, RCSI, Dublin, Ireland; 35 FutureNeuro SFI Research Centre, RCSI, Dublin, Ireland; 36 Department of Tumor Biology, Institute for Cancer Research, Oslo University Hospital, Oslo, Norway; 37 Center for Bioinformatics, Department of Informatics, University of Oslo, Oslo, Norway; 38 Center for Molecular Medicine, Department of Clinical Neuroscience, Neuroimmunology Unit, Karolinska Institutet, Stockholm, Sweden; 39 Institute of Environmental Medicine, Karolinska Institutet, Stockholm, Sweden; 40 Institute of Biological Psychiatry, Copenhagen Mental Health Services, Copenhagen, Denmark; 41 Danish Headache Center, Department of Neurology, Copenhagen University Hospital, 2600 Glostrup, Denmark; 42 Department of Clinical Medicine, University of Copenhagen, Copenhagen, Denmark; 43 The Lundbeck Foundation Initiative for Integrative Psychiatric Research, iPSYCH, Copenhagen, Denmark; 44 The Globe Institute, Lundbeck Foundation Center for Geogenetics, Øster Voldgade 5-7, 1350 Copenhagen K, Denmark; 45 Department of Zoology, University of Cambridge, Cambridge CB2 3EJ, UK; 46 Department of Integrative Biology, University of California, Berkeley, 3060 Valley Life Sciences Bldg #3140, Berkeley, CA 94720-3140, USA; 47 Faculty of Medicine, University of Iceland, Reykjavík, Iceland

**Keywords:** plague, second plague pandemic, Yersinia pestis, pandemic genomics, population genomics, selection, population replacement, Trondheim

## Abstract

Human populations have been shaped by catastrophes that may have left long-lasting signatures in their genomes. One notable example is the second plague pandemic that entered Europe in ca. 1,347 CE and repeatedly returned for over 300 years, with typical village and town mortality estimated at 10%–40%.^1^ It is assumed that this high mortality affected the gene pools of these populations. First, local population crashes reduced genetic diversity. Second, a change in frequency is expected for sequence variants that may have affected survival or susceptibility to the etiologic agent (*Yersinia pestis*).^2^ Third, mass mortality might alter the local gene pools through its impact on subsequent migration patterns. We explored these factors using the Norwegian city of Trondheim as a model, by sequencing 54 genomes spanning three time periods: (1) prior to the plague striking Trondheim in 1,349 CE, (2) the 17^th^–19^th^ century, and (3) the present. We find that the pandemic period shaped the gene pool by reducing long distance immigration, in particular from the British Isles, and inducing a bottleneck that reduced genetic diversity. Although we also observe an excess of large F_ST_ values at multiple loci in the genome, these are shaped by reference biases introduced by mapping our relatively low genome coverage degraded DNA to the reference genome. This implies that attempts to detect selection using ancient DNA (aDNA) datasets that vary by read length and depth of sequencing coverage may be particularly challenging until methods have been developed to account for the impact of differential reference bias on test statistics.

## Highlights

• The second plague pandemic homogenized ancestry in Trondheim
• Gaelic ancestry is sharply reduced in post-pandemic Trondheim
• Pervasive reference bias taints frequency differences observed between populations

## Teaser

Gopalakrishnan et al. investigate the genomic signatures of the second plague pandemic on the residents of Trondheim in Norway. They find that the pandemic resulted in a sharp reduction in Gaelic ancestry and also find evidence of differential reference bias among their ancient samples, which reduces the reliability of selection analyses.

## Results and discussion

**Figure 1:**
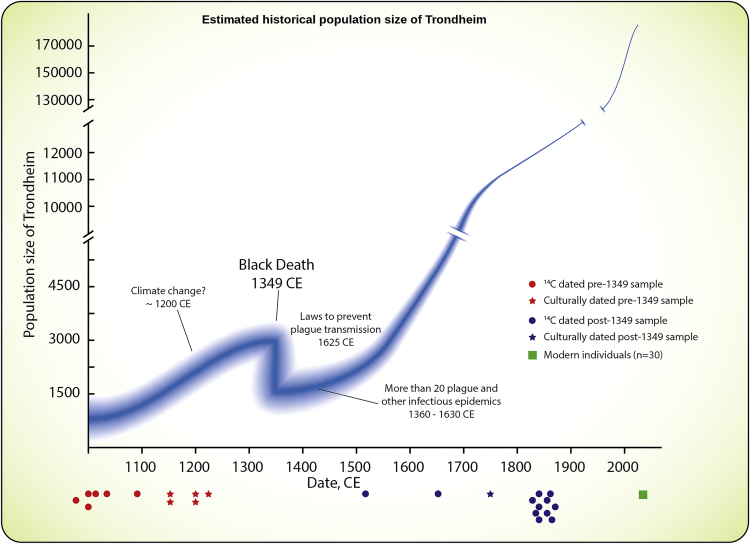
Estimated population size in Trondheim Illustrative reconstruction of Trondheim’s population size from 1,000 CE (based on Sandnes and Salvesen,^5^ Christophersen,^7^ and Sandnes and Supphellen^9^). Three major demographic phases were experienced: (1) an initial growth period up until around the 14th century, (2) a plague pandemic induced bottleneck followed by a low, fluctuating population size for ca. 300 years up to the mid-17th century, and (3) relatively rapid growth from ca. 1,650 CE. The midpoint of the estimate of the ages of samples from which genomic data were generated is shown on the x axis—for full age distributions see Table S1. See also Figure S1 and Data S1A.

It is now possible to directly assess the impact of catastrophic events, such as the second plague pandemic, on a population’s gene pool, by comparing genomes from individuals who lived before and after them. A key problem for such studies is the availability of sufficient numbers of adequately preserved remains from appropriate geographic locations that span the time window of interest. The medieval skeletal record from what is today the current Norwegian city of Trondheim is ideal for this purpose.^3^ The social and demographic impact of the second plague pandemic is well documented in Norway. Estimates of the number of deserted farms indicate that, around 1,500 CE, the Norwegian population was reduced to about 40% of its medieval pre-plague maximum^4,^ (Figure 1), and similar scale reductions are believed to have also affected cities such as Trondheim.^6^ Following its establishment by at least 970–980 CE,^7^ Trondheim grew rapidly until the plague first struck in 1,349 CE.^7^ Although the immediate impact of the initial attack is unknown, a number of documented plague epidemics followed until the first decades of the 17^th^ century.^8^ These epidemics reduced the city’s population,^6^ so that it did not regain its pre-plague size until the 17^th^ century^7^ (Figure 1). From the 18^th^ century, Trondheim’s population increased rapidly, to its current census size of around 180,000 inhabitants.

We used Illumina technology to sequence the genomes of 24 human skeletons excavated from two geographically close cemeteries from Trondheim (Table S1). One is the “Library site” (just north of the ruins of St. Olav’s church), that contains samples (n = 11) that date confidently to before 1,349 CE (the year the plague arrived in Trondheim). The other is the West Front of the Nidaros Cathedral, which contains samples (n = 13) that can be dated to between the 17^th^ and 19^th^ centuries. Archaeological and historical evidence suggest that both sites were parish churchyards that accepted burials from all socioeconomic backgrounds,^10^ making them ideal for assessing the impact of the plague on the Trondheim gene pool. The skeletons yielded whole-genome sequencing data at ca. 0.06–8.11× autosomal depth of sequencing coverage (DoC) (median = 1.75). To obtain a third temporal sample from Trondheim, we sequenced the genomes of 30 anonymous random modern inhabitants to ca. 30× DoC (Table S2; Data S1).

**Figure 2:**
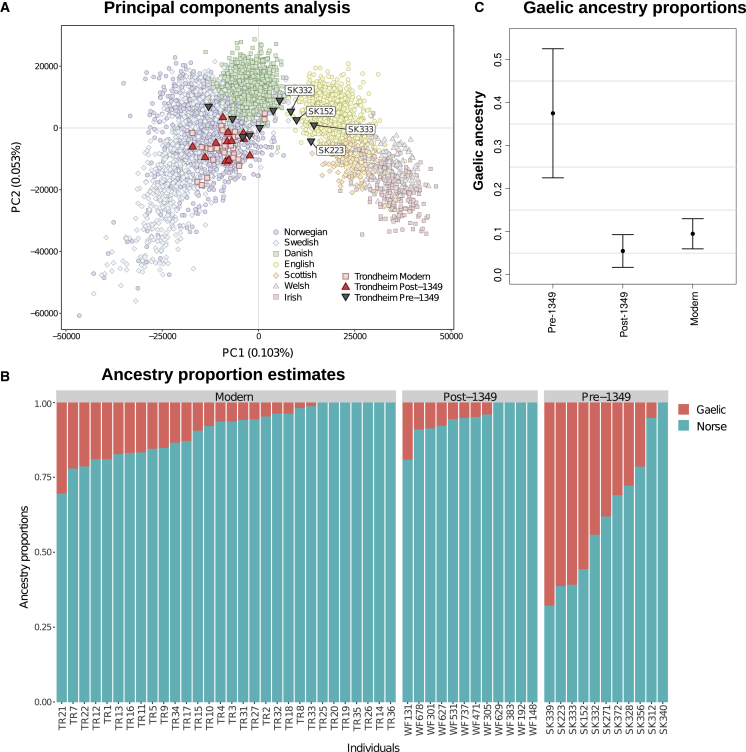
Ancestry estimates of ancient and modern Trondheim individuals (A) Principal-component analysis projection of the ancient and modern Trondheim individuals on the principal components estimated in reference populations from Scandinavia and the British Isles. (B) Ancestry proportions estimated for ancient and modern Trondheim samples, visualized using a supervised ADMIXTURE analysis with modern Norse and Gaelic populations used as the two reference populations. The high proportion of Gaelic ancestry in SK339 can likely be attributed to its low sequencing coverage. (C) The mean Gaelic ancestry of the three temporal groups from Trondheim, based on the ADMIXTURE results with 95% CI shown as error bars. See also Figures S2 and S3.

Validation of the palaeogenomic sequence data (Figure S1) revealed more DNA deamination in the samples from the older “Library site,” consistent with the expectations of the thermal age hypothesis.^11^ We next explored the relationship between the three temporal groups (pre-1,349, post-1,349, and modern) to each other (Figure S2) and several contemporary European reference populations. Projections of ancient individuals onto principal-component analysis (PCA) plots (Figures 2A and S3A) show that the post-1,349 and modern Trondheim individuals overlap and cluster within contemporary Scandinavians. While the pre-1,349 samples also cluster primarily with contemporary Scandinavians, they show little overlap with the two later groups. Furthermore, four individuals from the pre-1,349 group (SK152, SK223, SK332, and SK333) show evidence of ancestry from the British Isles. To explicitly test for individual differences in ancestry before and after the plague, we calculated D-statistics^12^ of the form D(YRI, X; Norse, Gaelic), where YRI are the Yoruba from Nigeria from the 1000 Genomes project,^13^ and the latter two groups are the chip-typed contemporary individuals from Norway and Sweden (Norse, n = 2,138), on the one hand, and Ireland and Scotland, excluding Orkney (defined here as Gaelic, n = 459), on the other. These analyses revealed that while most of the pre-1,349 and all post-1,349 samples appear to share more drift with Scandinavians, three out of the four above mentioned pre-1,349 samples exhibit closer relationship to the British Isles populations (Figure S3B). This conclusion is supported by direct estimates of ancestry obtained using ADMIXTURE in supervised mode, with the same reference populations (Figures 2B and 2C), which reveal that the pre-1,349 group had more Gaelic ancestry than both the post-1,349 (p = 0.00083) and the contemporary Trondheimers (p = 0.0021). Tentative further support comes from an analysis of dental strontium and oxygen isotopes based on enamel sampled from a subset of the samples. Specifically, while all individuals studied have combined isotope values that are consistent with Norwegian origins (Data S1; Figure S3C), the oxygen isotope value for one of the above mentioned pre-1,349 individuals (SK223) and the strontium isotope values for all four of above mentioned individuals are also consistent with many regions in the northern British Isles^14^^,^^15^^,^^16^ and thus could indicate that these individuals were raised there and later migrated to Trondheim.

Our findings indicate that Trondheim was a relatively cosmopolitan city prior to the second plague pandemic, attracting immigrants from not only local populations in Norway but much further afield,^17,^ consistent with claims that it was one of the major political, economic, and religious centers of Europe at that time.^9,^ Historical sources indicate that this status was drastically changed by the pandemic.^18^ Our survey of the Trondheim gene pool before and after the pandemic supports this conclusion, insofar as it became less cosmopolitan. The notable impact of the pandemic on the Trondheim gene pool contrasts with results based on mitochondrial DNA (mtDNA) sequences from similarly temporally structured British and Danish cohorts.^19^ In part, this may be due to the lower resolution of mtDNA as a single uniparentally inherited locus. However, it may also reflect differences in relative importance of the locations pre- and post-plague. For example, while London remained an important city throughout the time period, and thus would have continually drawn migrants from across large distances, Trondheim’s status changed from a place of major importance prior to the epidemic, to a relative backwater subsequently. Overall, our results suggest that if migrants entered Trondheim following 1,349 CE—whether pilgrims following St. Olav’s Way to the Nidaros cathedral,^20^ or tradesmen and workers to replace those lost, they were principally drawn from surrounding regions.

Noting the marked difference in ancestry between the pre- and post-1,349 Trondheim groups, we next sought to assess the demographic impact of the plague by comparing the nucleotide diversity (π, Figure S4) of the three temporal groups from Trondheim. Nucleotide diversity was computed in 500-kb windows across the genome using genotype likelihood in ANGSD.^21^ The average nucleotide diversity was highest in the modern samples, while the post-1,349 samples showed lower average nucleotide diversity across the genome than the pre-1,349 samples, as would be expected following a strong bottleneck (Mean π in 500-kb windows: pre-1,349: 211.09, post-1,349: 198.47, modern: 329.94; t test pre-1,349 versus post-1,349 p value < 10^−15^), which may in part be accounted for by the loss of Gaelic ancestry.

**Figure 3:**
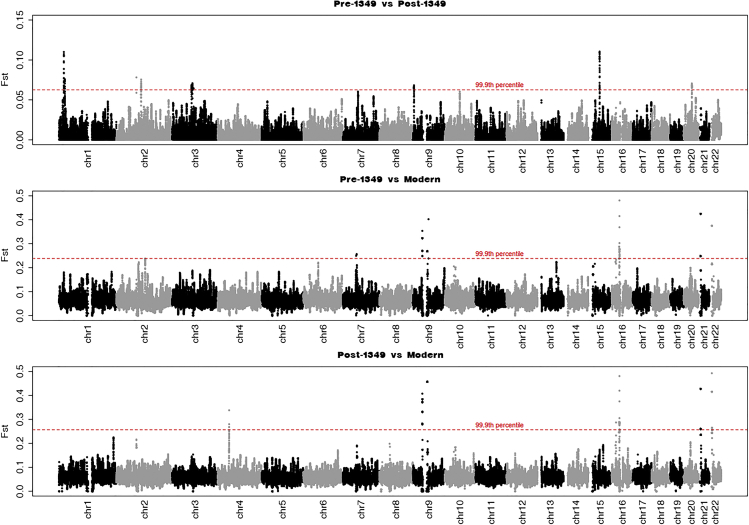
Population-pairwise F_ST_ estimates The Bhatia-Hudson estimate of F_ST_ (uncorrected for reference genome bias) for each of the two-way population comparisons, computed across the entire genome in 500-kb overlapping windows, using a step size of 20 kb. See also Figure S4.

**Figure 4:**
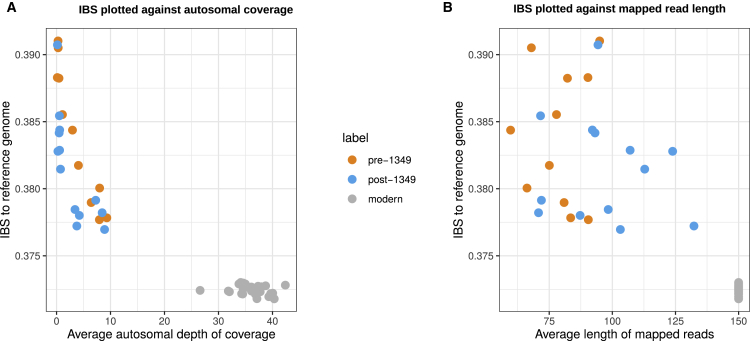
Reference bias among the historical samples The proportion of sites that are shared identical-by-state (IBS) to reference genome plotted as a function of sample characteristics. (A) The IBS values are plotted against the average autosomal sequencing depth of the samples. (B) The IBS values are plotted as a function of the length of mapped reads, for ancient and modern Trondheim individuals.

We used ANGSD^21^ to calculate the weighted average of the Bhatia-Hudson^22^ F_ST_ estimator for each of the two-way group comparisons, by scanning across the entire genome over 500-kb windows, using 20-kb steps (Figure 3). Although there was a significant difference between the pre- and post-1,349 groups (F_ST_ = 0.0155, SE = 0.0049), much greater differences were observed between either ancient group and the present (F_ST_ = 0.046 [SE = 0.016] for the pre-1,349 and F_ST_ = 0.049 [SE = 0.018] for the post-1,349). These results were qualitatively different from those of the PCA (Figure 2A), which indicated the greatest difference between the pre-1,349 group and the others. We postulated that the F_ST_ values could be affected by a bias of mapped reads from the ancient Trondheimers toward the allele represented in the human reference sequence—hereafter referred to as reference bias.^23^^,^^24^^,^^25^^,^^26^ This would result in underestimation of F_ST_ between ancient groups, and overestimation of F_ST_ between ancient and modern groups, but would have less impact on eigenvector values for ancient samples obtained from projection onto a PCA based on genotypes from contemporary individuals. To measure the reference bias, we calculated a simple identity-by-state (IBS) score between the pseudo-haploid genotypes of each Trondheim individual and the reference sequence. These results show a clear reference bias (greater IBS values) affecting the ancient individuals that is weakly correlated with read length (r = −0.17), but very strongly with log_10_ DoC (r = −0.87) (Figures 4A and 4B). Reference bias was strongest in those with low (<2×) coverage but remains markedly higher than in modern individuals even at 5×. Further analyses revealed a strong bias toward a higher frequency of the reference allele in the ancient groups for positions with high F_ST_ values when compared with modern Trondheimers (Figures S4B and S4C). This shows that the absolute values of F_ST_ are affected by reference bias and cannot be taken at face value. Accordingly, it is extremely challenging to determine the extent to which allele frequencies in Trondheim were affected by selection due to differential mortality from the plague. Based on these results, it is possible that reference bias will represent a significant challenge for studies based on ancient samples with divergent average read lengths and DoC, in particular when DoC is lower than 5× coverage. Until methods are developed to account for reference mapping bias, F_ST_ results based on comparing ancient and modern samples must be taken with extreme caution, unless the average number of reads per position for diploid chromosomes is sufficient to ensure that reads from both homologues have been encountered.

In conclusion, we show that the second plague pandemic had a notable impact on the Trondheim gene pool. From the Viking age and until the plague, Trondheim’s population contained ancestry from the British and Irish Isles^27^ but afterward, the diversity in ancestry seems to have been lost, presumably due to a loss of political and economic status. Due to the problem of reference bias described in this manuscript, we were unable to identify differences in allele frequencies before and after the plague caused by selection, which are also likely to have been affected by the loss of British and Irish ancestry. It is important to point out that this does not mean that no selection occurred in Trondheim as a result of the plague. Interestingly, a recent study that applied target capture sequencing to characterize 488 immune related genes from a cohort of plague pit versus modern residents of Ellwangen, Germany, reported evidence of allele frequency changes consistent with selection.^28^ It is possible that their results were not affected by reference bias in the way we demonstrate here. However, it would be prudent to explore the impact of reference bias in this instance and other such studies based on ancient DNA.

Overall, we believe that the key finding of our study relates to the technical challenge of reference bias affecting the mapping of sequence reads from low coverage ancient samples. We demonstrate that scans for positions affected by natural selection based on F_ST_ values are heavily affected by reference bias in the ancient individuals from Trondheim. Interestingly, projection of ancient samples onto PCAs generated using genotypes from contemporary individuals are less affected by reference bias,^23^^,^^24^^,^^25^ but as paleogenomic studies increasingly move their focus toward exploring how genomes have changed through time, they will need to find strategies to overcome the problem of reference bias. Studies that aim to detect selection using such datasets, for example, to test the hypothesis that host genetic factors may in part explain the observation that mortality ascribed to the second pandemic generally decreased through time, will only be possible if either computational methods are developed to reduce the introduction or impact of such biases, or larger and more deeply sequenced datasets are generated.

## STAR★Methods

### Key resources table

**Table:** 

REAGENT or RESOURCE	SOURCE	IDENTIFIER
**Biological samples**		

54 human blood or tissue samples: bones and teeth from 24 ancient samples, blood from 30 contemporary samples	This paper; See Data S1	N/A

**Chemicals, peptides, and recombinant proteins**		

Proteinase K	Sigma-Aldrich	Cat#3115844001
Phenol	Bionordika	Cat#A0447,0500
Chloroform	Sigma-Aldrich	Cat#288306-1L

**Critical commercial assays**		

Mycoarray human genome bait set	This manuscript	https://arborbiosci.com/genomics/targeted-sequencing/mybaits/mybaits-custom-dna-seq/
DNeasy Blood & Tissue Kit	QIAGEN	Cat#69506
MinElute PCR Purification Kit	QIAGEN	Cat#28006
NEBNext DNA Sample Prep Master Mix Set 2	New England Biolabs Inc.	Cat#E6070

**Deposited data**		

Whole genome sequencing of 54 modern and ancient samples from Trondheim, Norway	This manuscript	ENA project PRJEB53899; ENA samples ERS12286163 – ERS12286216
Genotypes for CEU individuals from 1000 genomes	1000 Genomes Project Consortium^13^	Population label CEU
2139 contemporary Europeans	Ebenesersdóttir et al.^51^	https://www.science.org/doi/10.1126/science.aar2625
Human reference genome hg38	Genome Reference Consortium	https://www.ncbi.nlm.nih.gov/assembly/GCF_000001405.26/

**Software and algorithms**		

AdapterRemoval2	Schubert^46^	https://adapterremoval.readthedocs.io/en/stable/index.html
Bwa	Li and Durbin^47^	http://bio-bwa.sourceforge.net/
Picard		https://broadinstitute.github.io/picard/
Samtools	Li et al.^48^	http://www.htslib.org/
Bedtools	Quinlan and Hall^49^	https://bedtools.readthedocs.io/en/latest/
mapDamage 2.0	Jónsson et al.^50^	https://ginolhac.github.io/mapDamage/
ANGSD	Korneliussen et al.^21^	http://www.popgen.dk/angsd/index.php/ANGSD
ngsRelate	Hanghøj et al.^63^	https://github.com/ANGSD/NgsRelate
smartPCA	Patterson et al.^12^	https://github.com/DReichLab/AdmixTools
ADMIXTOOLS	Patterson et al.^12^	https://github.com/DReichLab/AdmixTools
ISOGG	Poznik et al.^53^ and Karmin et al.^54^	https://isogg.org/tree/

### Resource availability

#### Lead contact

Further information and requests for resources and reagents should be directed to and will be fulfilled by the lead contact, Shyam Gopalakrishnan (shyam.gopalakrishnan@sund.ku.dk).

#### Materials availability

This study did not generate new unique reagents.

### Experimental model and subject details

The current study uses short read sequencing data from the full genomes of 24 historic skeletons and 30 anonymously sampled modern residents of Trondheim. Ethical approval to generate palaeogenomic data from the ancient samples was provided by the Norwegian National Committee for Research Ethics on Human Remains (Nasjonalt utvalg for vurdering av forskning på menneskelige levninger), reference 2011/73. The modern DNA was derived from blood originally provided under appropriate ethical review to the Biobank1 at the Sankt Olav Hospital, Trondheim for use in research purposes. All information on the context and sequencing coverage of the samples is provided in the STAR Methods and Tables S1 and S2.

### Historical and archaeological context

The settlement that today is known as the modern day city of Trondheim was founded by ca. 970–980 CE as a trading post, and its strategic position made it a prominent link to the sea, and thus Europe, for the regional community. With regards to the terminology that we use, we highlight that although for consistency we use the term ‘city’ throughout our manuscript when referring to both the modern and historic settlements in Trondheim, in general historical and archaeological scholarly discussions about medieval urban communities in the Nordic countries typically refer to them as ‘towns’, as a parallel to old norse: ‘kaupstadr’ and ‘kaupbær’. Trondheim became a site of pilgrimage for Christians following the canonisation of its founder and the establishment of the powerful Nidaros Cathedral in 1152 CE, along with thirteen other churches in the city centre.^29^ Also named Nidaros during the Middle Ages, the city was the capital of Norway until 1217 CE. The surrounding area, called Sor-Trøndelag, is still a fertile district that is well adapted to agriculture, and able to sustain both a rural and town population during the medieval period. However this period of prosperity was short lived. A marked decline dating from 1275 CE is indicated through a dramatic decrease both in the number of buildings and of finds of all sorts.^30^ The impact of the Reformation,^31^^,^^37^ the loss of the archdiocese of Norway following the introduction of Lutheran Protestantism in 1537 CE, and overall economic decline impacted the city’s status. This was further intensified with the increasing fish trade monopoly building up in Bergen^32^ as well as the restrictive trade decree, enacted in 1384 CE.^33^ It is thought that Trondheim’s industries at the time remained underdeveloped, and could not compete with the more technologically advanced centers elsewhere.^34^ The lack of building activity in the 14th century is thought to be linked to the destructive impact of the second plague pandemic,^35^ and subsequent catastrophic fires wreaked havoc, particularly in 1651 and 1681 CE. Following these events, the city was replanned and rebuilt,^36^ making the assessment of early medieval building distribution difficult.

Outbreaks of disease were common, with the notable bubonic plague thought to have arrived in the city in 1349 CE, with a high mortality cost.^33,^ Documented outbreaks of plague struck in 1370, 1566, 1599-1601, 1618 and 1629 CE. In 1600 CE, a chronicler stated that over 1800 people died of plague in Trondheim. As many as 978 people were said to have been buried in 1629-30 CE.^8^ Moreover, frequent outbreaks of smallpox affected the population, and all evidence points to impoverished and poor populations from the 14th century onwards.^33^ This was further exacerbated by the effect of the Little Ice Age. However, Trondheim endured, and today it is the fourth largest city of Norway, with a rich archaeological record of the past millennium. Large parts of the city’s centre have been excavated in connection with urban renewal over the last 150 years. This provides a remarkable insight into the last 1000 years of the city’s history, including knowledge about living conditions, housing, fires, churches and churchyards. Among these excavations were several churchyards, resulting in skeletal material from ca. 5000 individuals that are now stored at the NTNU University Museum.^38^ The medieval skeletal collections have been the subject of several studies investigating health,^38^^,^^39^ e.g. the possible traces of the bacterial pathogen associated with endemic syphilis,^40^ premaxillary hyperdontia,^41^ and osteoporosis,^42^ as well as the origin and mobility of people living and dying in medieval and post-medieval Trondheim. In this regard stable oxygen isotope data indicated a high degree of mobility pertaining to the medieval population of Trondheim.^10^

For the present study skeletons from two cemetery collections were utilised, the West Front (Nidaros Cathedral churchyard) and the Library Site (St Olav’s Site). Together these cemeteries span a use of ca. 800 years of occupation of the city of Trondheim.

The first of these is the West Front burial ground of the Nidaros cathedral (samples labelled WF), that was excavated in the mid 1990s, yielding skeletal remains of 60 individuals: 21 males, 22 females and 17 with uncertain sex. At the time of excavation, it was noted that the preservation of the materials was excellent, as most of the graves were embedded in clay (an anaerobic environment). The churchyard is known to have been in use from 1585 CE when the cathedral became a parish church, and until the closing of the churchyard in 1897 CE.^39^ As such the samples can be confidently assigned as dating to after the first arrival of the plague pandemic - something we subsequently confirmed for most of the samples using AMS ^14^C dating of associated skeletal elements (Table S1).

The second burial ground, known as the Library Site, was located just north of the ruins of St Olav’s church, and is believed to have been its churchyard (samples labelled SK). This latter site was excavated between 1973-85 CE in relation to the construction of a new public library, and was originally dated using artefact associations to have been in use from the beginning of the 12th century until the 17th century. A total of 389 skeletons were found; 133 males, 126 females and the remainder with uncertain sex. The skeletal material is generally well preserved and even some brains and spinal columns were recovered (but since then damaged, due to electrical failure). However, the skeletons from the southern part of the burial ground closest to the church, were buried in gravelly sand, and have consequently bad preservation.^38^ For this study, we specifically chose specimens that fall into the earliest site phases, and thus believed to date to the earliest part of the occupation (thus pre-pandemic). This was subsequently confirmed for most of the specimens using AMS ^14^C dating of associated skeletal elements (Table S1).

### Method details

#### Demographic history reconstruction

Figure 1 represents an illustrative reconstruction of the census population size of Trondheim from ca. year 1000 CE, based on archaeological findings^5^^,^^6^^,^^7^^,^^9^ and for the latter period, the official demographic statistics.^43^ Estimates from the period 1000 – 1349 CE are based on Sandnes,^9^ and references therein, Christophersen,^7^ and Christophersen and Nordeide.^6^ It is unclear exactly how many people lived in the area at the time of the city’s foundation, but estimates have been made that it could have been in the range of a few hundred.^7^ Sandnes^9^ provides an estimated population size at the end of the 13^th^ century, and both Christophersen^7^ and Christophersen and Nordeide^6^ provide information regarding relative population size changes in the given time period, as well as the estimate of 70% reduction of population size in 1349 CE. Data in the period 1349 – 1769 CE is taken from Sandnes and Salvesen^5^ and Sandnes et al.^9^ Temporal fluctuations in population size are partly inferred from the occurrences of plague epidemics in the period,^8^ even though it is difficult to be certain whether epidemics of the time are due to plague or some other infectious disease. From 1769 CE official demographic statistics were used.^43^

#### Ancient DNA methods

##### Laboratory methods

DNA was prepared for palaeogenomic sequencing in the dedicated clean laboratory facilities at the Centre for GeoGenetics, Natural History Museum of Denmark. DNA was principally extracted from either whole tooth roots or femur samples. The external face of the samples were initially pre-cleaned with dilute bleach solution, prior to powdering. DNA extractions were performed using a silica in-solution approach that is optimised for large volumes of powder, following,^44^ prior to conversion into Illumina compatible sequencing libraries,^45^ that in brief incorporates a blunt end adaptor ligation step, prior to PCR amplification of the library. Post amplification, a subset of the libraries were enriched by whole-genome capture using Mycroarray LLC’s commercially available human genome bait set, following the manufacturer’s guidelines (Data S1). The amplified libraries were subsequently sequenced at the Danish National High-Throughput DNA-Sequencing Centre on an Illumina HiSeq 2500 using SR100 chemistry, and by deCODE genetics on Illumina MiSeq (PE150) and Illumina HiSeq 2500 (PE100) platforms (Data S1). To complement the palaeogenomic data, we also resequenced the genomes of 30 anonymised modern Trondheim inhabitants (Data S1). This DNA was derived from blood originally provided under appropriate ethical review to the Biobank1 at the Sankt Olav Hospital, Trondheim for use in research purposes. Each genome was sequenced on Illumina X10 platforms to ca. 30x coverage using Novogene’s commercial service.

##### Bioinformatic processing

Nucleotide base calling and quality score assessment in the sequence data was performed using specific Real Time Analysis (RTA) software provided by Illumina. As the first step of the pipeline, low quality and missing bases were trimmed from the reads, followed by removal of adapters using AdapterRemoval2.^46^ Subsequently, the reads from each sample were mapped to the human genome build38 (v0.7.10; aln algorithm),^47^ with the seed disabled (-1024) to improve accuracy and the minimum base quality set to 15. The HiSeq and MiSeq mapped reads were merged at the library level and subsequently filtered for PCR and optical duplicates using Picard (v1.79, https://broadinstitute.github.io/picard), and reads that mapped to multiple locations in the genome were excluded. Bases with base qualities lower than 20 and reads with mapping quality lower than were discarded using samtools (v1.5).^48^ Read depth and coverage were determined using bedtools-2.18.2^49^ and an inhouse python script. Finally, base quality scores were rescaled with MapDamage2.0^50^ to exclude likely-damaged bases.

### Quantification and statistical analysis

#### Quality control

##### Data validation

We used several approaches to validate the dataset. Firstly, all libraries yielded short read lengths (Data S1) and patterns of cytosine deamination as visualised with MapDamage2.0^50^ were consistent with aDNA expectations (Figure S1). Secondly, we estimated DNA contamination based on the mtDNA and the X-chromosome ('Contamination' program in ANGSD^51^ v.0.911) for individuals with one X-chromosome as described in Ebenesersdóttir et.^51^ The results (Data S1) indicate that while there is some evidence of contamination in the DNA extracts, it is not at a depth sufficient to affect the overall results. We considered samples (with one X-chromosome) to be contaminated if they showed contamination for both X-chromosome and mtDNA. After calling consensus mtDNA sequences, we evaluated whether the minor alleles were more likely to be due to contamination, or errors in sequencing, by looking at the frequency distribution of the minor alleles and whether or not they matched known mtDNA haplogroups (see Data S1). Thirdly we used R_Y_^52^ to sex each sample (Table S2) and obtained clear assignments for the all samples except SK531.

##### Estimation of relatedness

To investigate if any of the Trondheim samples in this study are relatives, we estimated the relatedness coefficients for all pairs of individuals within each of the three Trondheim datasets: pre-plague samples (sk), post plague (wf) samples and modern (tr) samples. Due to the low sequencing depth of coverage of the historical samples, we performed the estimation with the software ngsRelate,^63^ which takes the uncertainty of genotypes inherent to low coverage data into account by basing the estimates on genotype likelihoods. Also, since it can be difficult to confidently identify polymorphic loci from datasets with a low number of samples that are only sequenced to low-depth, like sk and wf, we based the estimation only on data from loci that were polymorphic (minor allele frequency (MAF) above 0.05) in 99 individuals from the population with Northern and Western European ancestry (CEU) from the 1000 Genomes Project phase 3.^13^ As input for ngsRelate, we used genotype likelihoods calculated with the samtools model implemented in ANGSD.^20^ Furthermore, we used allele frequencies estimated in several different ways to make sure the results are robust to the way the allele frequencies are estimated. Specifically, for the two historical datasets, pre-1349 and post-1349, we 1) estimated allele frequencies directly from the sequencing data from the samples in the dataset itself using ANGSD, 2) estimated allele frequencies from the CEU population (99 individuals) and 3) estimated allele frequencies from the sequencing data from the modern samples (tr) using ANGSD. Similarly, we used two sets of allele frequencies for the modern dataset (tr): we 1) estimated allele frequencies directly from the sequencing data from the samples in the dataset itself using ANGSD and 2) estimated allele frequencies from the CEU population. For all the datasets, only reads with mapping quality above 30 and base quality above 20 were included and only transversion SNP sites were used for the analyses of the two historical datasets, since transition sites are known to have increased error rates in historical samples. Finally, before applying ngsRelate we filtered away sites with MAF below 0.05 in each of the datasets, this led to datasets with the number of SNPs ranging from ∼1 million (pre-1349 with allele frequencies based on the dataset itself) to ∼6 million (modern trondheim individuals). ngsRelate was applied ten times to each combination of datasets and estimates of allele frequencies to be able to assess if the underlying maximum likelihood algorithm reached convergence. This resulted in ten maximum likelihood estimates of the relatedness coefficients k_0_, k_1_ and k_2_, for each pair of individuals within each dataset, where k_x_ is the proportion of the genome where the pair shares x alleles identical by descent. For each pair, the difference in log likelihood between the runs with the 5 highest log likelihoods was assessed and found to be below 0.1 log likelihood units, suggesting convergence was reached for all pairs.

The estimates for pairwise relatedness from the run with the highest log likelihood for each dataset with different allele frequencies are shown in Figure S2. Although the estimates for the same dataset are affected by the different strategies used to estimate allele frequencies, the pair of individuals with the highest estimated k_1_ and k_2_ values is a pair of modern samples (tr13 and tr27) that was estimated to have a k_1_ of 0.155 (based on the allele frequency of modern samples) or 0.26 (based on the allele frequency of CEU), which is between what is expected for first cousins (E(k_1_)=0.25) and cousins once removed (E(k_1_)=0.125). Hence, across all the datasets, the samples in general do not seem to be closely related.

#### Population genomics

##### Population genomics analyses

Due to the low sequencing coverage depth of the ancient samples, haploid genotypes were obtained by randomly sampling one allele from reads with high-quality (Q≥30) base calls. SmartPCA^12^ was used to perform an initial principal components analysis (PCA) against a broad SNP chip dataset of 2,139 contemporary Europeans sampled across 28 populations, genotyped on microarray SNP chips^51^ from which 227,056 SNPs have been genotyped (Figure S3A). We repeated this analysis using reduced datasets of Scandinavian and British Isles populations (Figure 2A) to obtain a higher resolution window into the relationship of the ancient samples with closely related populations.

We used two other approaches to more explicitly test the hypothesis that the Trondheim population received gene flow from a wider geographic area prior to the second pandemic. Firstly, we grouped the contemporary genotyped individuals into two wider population clusters, Scandinavian (Norway, Sweden) and British Isles Gaelic (Irish and Scottish excluding Orkney), then used D-statistics as implemented in ADMIXTOOLS^12^ (using the Yoruba from Nigeria [YRI] as an outgroup) to visualise the relationship between each ancient Trondheim individuals and the two reference populations (Figure S3B). This observation of British Isles influence prior to the second pandemic is retained when we obtained more direct estimates of ancestry for each ancient sample, using ADMIXTURE in supervised mode, with the same datasets as reference populations (at K=2, Figure 2B). Overall therefore, our findings are consistent with the hypothesis that Trondheim experienced considerable immigration from outside of Scandinavia in its early history, after which this largely ceased. The signal of British Isles ancestry is not present after the arrival of the second pandemic, providing evidence that this diversity was lost during the pandemic. This raises the question as to whether this was due to selection against British Isles genomic components, or stochastic processes associated with the population bottleneck followed by any population replacement that occurred coming principally from Scandinavia.

##### mtDNA and Y chromosome haplogroups

We used the method of Ebenesersdóttir et al. to call the mtDNA (Table S2)^51^ and (where relevant) Y chromosome haplogroups (Table S2) for each ancient and modern Trondheim individual. In total, 7,972,586 positions from accessible regions of the Y chromosome had been called. Using further filtering of haplogroup informative sites,^53^^,^^54^ https://isogg.org/tree/, https://www.yfull.com/tree/) we focused on 4,239 positions where at least one individual in our 25 ancient and modern Trondheim male samples carried a derived allele to determine the SNPs that were most uniquely associated with any given sample and their phylogenetic affiliation (Table S2). We found that haplogroups H, J, T and U for the mitochondria, and haplogroups I1a, R1a and R1b for the Y chromosomes were the most frequent, all of which are common and widely spread in Scandinavia today.^55^ The modern genomic data also clearly demonstrates how at least in recent times, the population of Trondheim has been affected by migration from further away. Specifically in the post-plague ancient and in the modern sample we observed mtDNA haplogroups V, U5b1b1a and Z, and Y chromosome haplogroup N characteristic to the Saami people who inhabit the far North of Scandinavia.^56^

#### Isotope analyses

Multiple isotope analyses (strontium, oxygen, and carbon) of dental enamel was conducted on samples from the Library (n=9) and West Front sites (n=13) in Trondheim to investigate childhood origins and identify possible first generation migrants. The isotope results (Figure S3C; Data S1 D) were compared to previously reported human isotope values from Trondheim,^57^ and human and bioavailable (animal and plant) isotope values from Norway.^57^^,^^58^^,^^59^

##### Laboratory methods

Enamel was extracted from 20 teeth from the Library (n=7) and West Front (n=13) sites. All aspects of the analyses were conducted by JEL author using standard protocols and procedures for the isotopic analyses of archaeological dental enamel (for details, see Laffoon^60^). Sample preparation and processing were conducted at the Laboratory for Archaeological Chemistry, Department of Archaeological Science, Leiden University and measurements of isotope compositions via mass spectrometry were conducted at the Laboratory for Isotope Geochemistry, Faculty of Science, Vrije Universiteit Amsterdam. Teeth samples were mechanically cleaned to remove the outer layer of surface enamel and to expose the inner core enamel. Approximately 2-4 mg of core enamel was extracted using a hand-held drill equipped with a pre-cleaned, diamond-tipped rotary burr. The drill bit was cleaned before and between each sample extraction to avoid cross-contamination. Extracted enamel samples were subjected to chemical pre-treatment following the protocol ofBocherens et al.^61^

For strontium isotope analysis, enamel samples were dissolved in 3N nitric acid (HNO3) and loaded onto cation exchange columns comprising Sr-specific crown ether resin for separation of strontium from the sample matrix. After separation, strontium samples were loaded onto pre-cleaned, degassed rhenium filaments and ^87^Sr/^86^Sr ratios were measured on a ThermoScientific Triton Plus multi-collector thermal ionization mass spectrometer (TIMS). Long term measurements of the standard reference material (NBS-987) produced a mean ^87^Sr/^86^Sr of 0.710250 ±0.00001 (1σ) and the typical analytical error for all samples reported here is <0.00001. The strontium yields of blanks are consistently low (<100 pg) and negligible relative to the overall amount of strontium in enamel samples.

Oxygen and carbon isotope compositions of the bioapatite (carbonate) component of dental enamel were measured on a Finnigan DeltaPlus Isotope Ratio Mass Spectrometer, following reaction of the carbonate sample with orthophosphoric acid (H_3_PO_4_) [100%] and isolation of the produced carbon dioxide (CO_2_) with a Gasbench II universal automated. Long term reproducibility of the standard reference material (NBS-19) for δ^18^O is <0.2‰ and for δ^13^C is <0.1‰. Measurement drift is additionally monitored through the analysis of an in-house carbonate standard (VICS-1). All δ^18^O and δ^13^C values referenced herein are reported in the delta (δ) notation, in parts per thousand (‰) relative to the international VPDB (Vienna Pee Dee Belemnite) standard.

##### Isotope results

The carbon isotope values from this study are somewhat variable ranging from -16.1 to -12.2‰ indicating inter-individual differences in seafood consumption but no significant difference between the earlier and later dated individuals (Data S1 D). The oxygen isotope results are also moderately variable ranging from -8.6‰ to -4.1‰ and with significant differences between the early and late samples with generally higher (less negative) values for the former (Figure S3C). While oxygen isotope results, in general, reflect climatic and geographic conditions, it can be difficult to disentangle spatial and temporal variation. The pre-1349 samples overlap chronologically with the so-called Medieval Warm Period (with warmer temperatures linked to higher δ^18^O values) and the post-1349 samples overlap considerably with the Little Ice Age (with cooler temperatures linked to lower δ^18^O values). This pattern was previously reported by Fricke and colleagues^62^ for Medieval populations from Greenland and Denmark. As such, the most parsimonious explanation for the observed pattern in oxygen isotope values at Trondheim is that the systematic differences between the earlier and later dated samples reflect climatic change between these periods. The strontium isotope ratios also display considerable variation ranging from 0.7092 to 0.7158 (Figure S3C). This range of variation exceeds what one would expect if the sample represented a single local population. Comparisons with previously reported isotope data from Trondheim^57^ suggest that while most individuals are consistent with a local origin, several individuals have non-local values for strontium isotope, oxygen isotopes, or both (Figure S3C). However, given the relatively small sample size of the comparative data set (n=9), it may not reliably reflect the complete range of local variation for Trondheim. Zooming out to the broader (national) scale, nearly all isotope results from this study fall within the overall range of isotope variation (Figure S3C) previously reported for Norway.^57^^,^^58^^,^^59^ Broadly speaking, in Norway, individuals with higher ^87^Sr/^86^Sr ratios and lower δ18O values are expected in more northerly/inland locales, while individuals with lower ^87^Sr/^86^Sr ratios and higher δ^18^O values are expected in more southerly/coastal locales. Lastly, several pre-1349 individuals were identified as having potentially higher levels of Gaelic ancestry based on genomic analysis (labelled in Figure S3C). While these individuals have combined isotope values that are consistent with Norwegian origins, their isotope values are also consistent with many regions in the northern British Isles.^14^^,^^16^ Thus although the results of this analysis do not contradict a derivation of some individuals from the northern British Isles, as the current baseline data regarding strontium isotopes are coarse in Norway, they overall limit the outcome of the present isotopic analysis, and this equifinality makes interpretations of the origins and identification of (first generation) migrants rather equivocal.

#### Loci putatively shaped by the second pandemic

##### Nucleotide diversity and heterozygosity in the three populations

We summarised the nucleotide diversity in the three Trondheim groups, ie. pre-1349, post-1349 and modern Trondheimers by computing the pairwise differences (π) in overlapping windows of 100 kb, with a step size of 10 kb, across the genome. We computed the pairwise differences using ANGSD (v0.921), setting minimum base quality to 20 and minimum mapping quality to 30 (Figure S4).

We then used F_ST_ as the primary statistic to identify regions of the genome that showed a high degree of differentiation between the pre-1349 and the post-1349 samples. Subsequently, we computed nucleotide diversity and heterozygosity in the three groups as a means of lending further support to the regions found using high F_ST_ values.

##### Masking regions of genome

Before performing any analyses to identify regions of the genome that could be under selection, we masked regions of the genome that might be prone to leading to spurious findings of high F_ST_, due to genome accessibility. To address this, we obtained the list of regions that were annotated by the 1000 genomes project^13^ to be outliers in terms of genome accessibility using their strict filtering criteria. These regions were excluded from all downstream analyses.

##### Pairwise F_ST_ computation

The fixation index, F_ST_, was computed for each of the three Trondheim groups in our dataset, i.e. pre-1349 vs post-1349, pre-1349 vs modern, and post-1349 vs modern. Since the coverages and the sample sizes varied widely among the three populations, we used genotype likelihood based approach, as implemented in angsd (v0.929) to compute pairwise F_ST_. Using the accessibility-masked regions of the genome, we computed the pairwise F_ST_ using angsd and the companion program realSFS. First, we obtained the site allele frequency files (saf) in angsd, using minimum base quality of 20 and minimum mapping quality of 30. Further, we discarded reads that had a flag greater than 255, and used the -C 50 and base alignment quality score computation options in angsd. Finally, we required that only sites where at least 80% of the samples were covered by at least one read were included in the analysis. Using these saf files, we computed the pairwise F_ST_ in realSFS using its implementation of the Hudson-Bhatia estimator of F_ST_, in windows of 500kb with a step size of 20kb (Figure 3).

#### Impact of reference bias on F_ST_ results

We used the following procedure to evaluate the impact of reference bias in the ancient samples on F_ST_ estimates. First, we explored how the Identity by State of the mapped reads to the reference genome (refIBS) behaved as a function of autosomal depth of sequencing coverage (Figure 4A) and read length (Figure 4B). These plots clearly show there is a negative correlation between IBS and both sequencing depth and read length. Restricting to the ancient samples, we found refIBS to be negatively correlated with read length (r=-0.17), and a particularly strong negative correlation with sequencing depth (Figure 4A, r=-0.87, with sequencing depth on the log scale). In a linear regression model, sequence depth (log transformed) and read length combined account for 89.7% of the variance in refIBS among the ancient samples. Next, for each pair of populations (pre-1349 v post-1349, pre-1349 v modern and post-1349 v modern), we used positions with F_ST_ values > 0.07 (calculated using angsd, as described previously in the methods), where there were at least three reads carrying the alternative allele across all individuals. For each such position, we calculated the read frequency of the reference allele for each individual and then the means across individuals for the two populations being compared. The statistic of interest is the difference between these means, Δf_*ref*_. In the absence of reference bias, a positive correlation between F_ST_ and abs(*Δf*_*ref*_) is expected, but no clear association should be found between F_ST_ and signed *Δf*_*ref*_ (because neither population is expected to be systematically closer to the reference sequence). Sequence data from ancient individuals is much more likely to be affected by reference bias than sequence data from modern individuals. We would therefore expect to see more asymmetry of *Δf*_*ref*_ when comparing ancient and modern groups, than when two ancient groups or two modern groups are compared. In Figure S4B, we show the relationship between F_ST_ and *Δf*_*ref*_ for the three pairs of populations in heatmaps for chromosome 1 (the same kind of results are observed for all chromosomes). A clear reference bias leading to inflated F_ST_ values can be seen when the pre-1349 (Pearson's r=0.399, p<2.2x10^-16^) and post-1349 (r=0.366, p<2.2x10^-16^) populations are compared with modern Trondheimers. Less asymmetry is seen when the ancient groups are compared with each other (r=0.097, p<2.2x10^-16^). However, as shown in Figure S4C, the pre-1349 sample has significantly greater frequency of the reference allele than the post-1349 sample, such that some inflation of F_ST_ values due to reference bias is also expected when the two ancient groups are compared, although greater inflation is expected for comparisons of ancient versus modern groups. Consequently, for all three pairs of populations, the F_ST_ values estimated for individual positions or genomic regions cannot be taken at face value.

## Data Availability

• The short read sequences used in this paper are available at the European Nucleotide Archive under the project accession PRJEB53899. Accession numbers are listed in the key resources table. • This paper does not report original code. • Any additional information required to reanalyze the data reported in the paper is available from the lead contact upon request.

## Supplemental information

Document S1. Figures S1–S4 and Tables S1–S3

Data S1. Sequencing information, contamination estimates, isotope analysis and census population sizes, related to STAR Methods and Figure 1 (A) Library preparation and sequencing information, and per-library sequencing yield/summary statistics, (B) sex chromosome based contamination analysis, (C) mitochondria based contamination analysis, (D) strontium and oxygen isotope analysis, and (E) census population size of Trondheim from 1769 to 2011.

Document S2. Article plus supplemental information
