# Supplementary material for: The population genomic legacy of the second plague pandemic
Source: Curr Biol. 2022 Nov 7;32(21):4743–4751.e6. doi: 10.1016/j.cub.2022.09.023 (PMC9671091; doi:10.1016/j.cub.2022.09.023)
Supplement: Document S1. Figures S1–S4 and Tables S1–S3 [file mmc1.pdf]

Current Biology, Volume *32*

Supplemental Information

The population genomic legacy of the second plague pandemic

Shyam Gopalakrishnan, S. Sunna Ebenesersdóttir, Inge K.C. Lundstrøm, Gordon Turner-Walker, Kristjan H.S. Moore, Pierre Luisi, Ashot Margaryan, Michael D. Martin, Martin Rene Ellegaard, Ólafur þ. Magnússon, Ásgeir Sigurðsson, Steinunn Snorradóttir, Droplaug N. Magnúsdóttir, Jason E. Laffoon, Lucy van Dorp, Xiaodong Liu, Ida Moltke, María C. Ávila-Arcos, Joshua G. Schraiber, Simon Rasmussen, David Juan, Pere Gelabert, Toni de-Dios, Anna K. Fotakis, Miren Iraeta-Orbegozo, Åshild J. Vågene, Sean Dexter Denham, Axel Christophersen, Hans K. Stenøien, Filipe G. Vieira, Shanlin Liu, Torsten Günther, Toomas Kivisild, Ole Georg Moseng, Birgitte Skar, Christina Cheung, Marcela Sandoval-Velasco, Nathan Wales, Hannes Schroeder, Paula F. Campos, Valdís B. Guðmundsdóttir, Thomas Sicheritz-Ponten, Bent Petersen, Jostein Halgunset, Edmund Gilbert, Gianpiero L. Cavalleri, Eivind Hovig, Ingrid Kockum, Tomas Olsson, Lars Alfredsson, Thomas F. Hansen, Thomas Werge, Eske Willerslev, Francois Balloux, Tomas Marques-Bonet, Carles Lalueza-Fox, Rasmus Nielsen, Kári Stefánsson, Agnar Helgason, and M. Thomas P. Gilbert

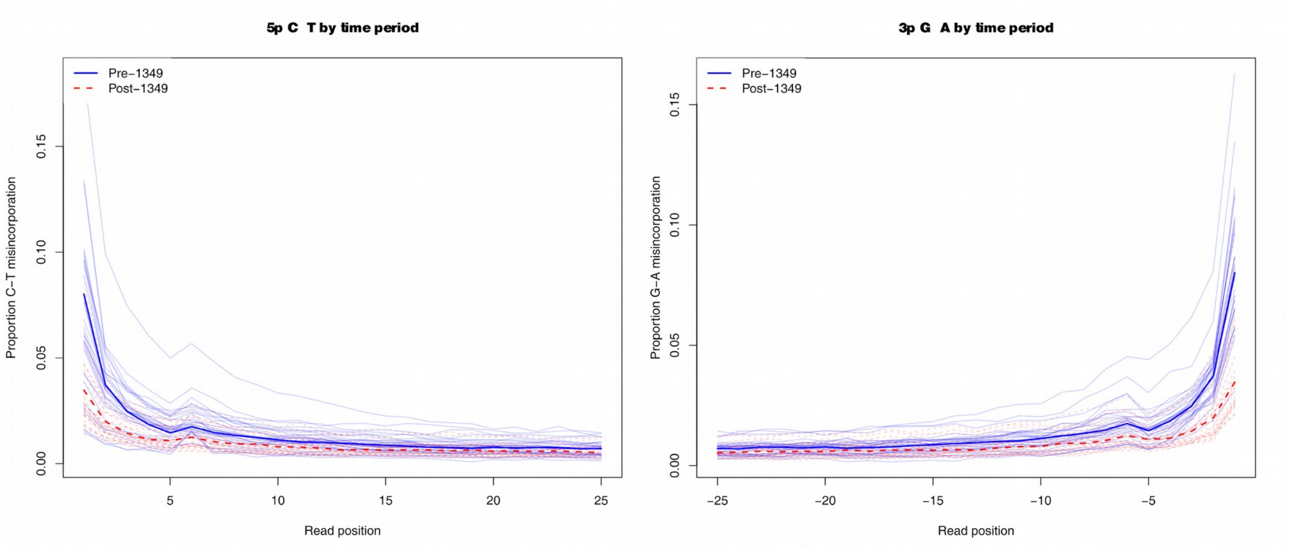

Figure S1. DNA damage profiles. Related to Data S1 A, Figures 1 and 4. 5' C-T misincorporation on the left, and 3' G-A misincorporation on the right, across the two time periods. The bold solid blue line denotes the mean for the pre-1349 samples, whereas the bold dashed red line denotes the mean for the post-1349 samples. The faded solid blue and dashed red lines are the misincorporation rates estimated for the individual samples.

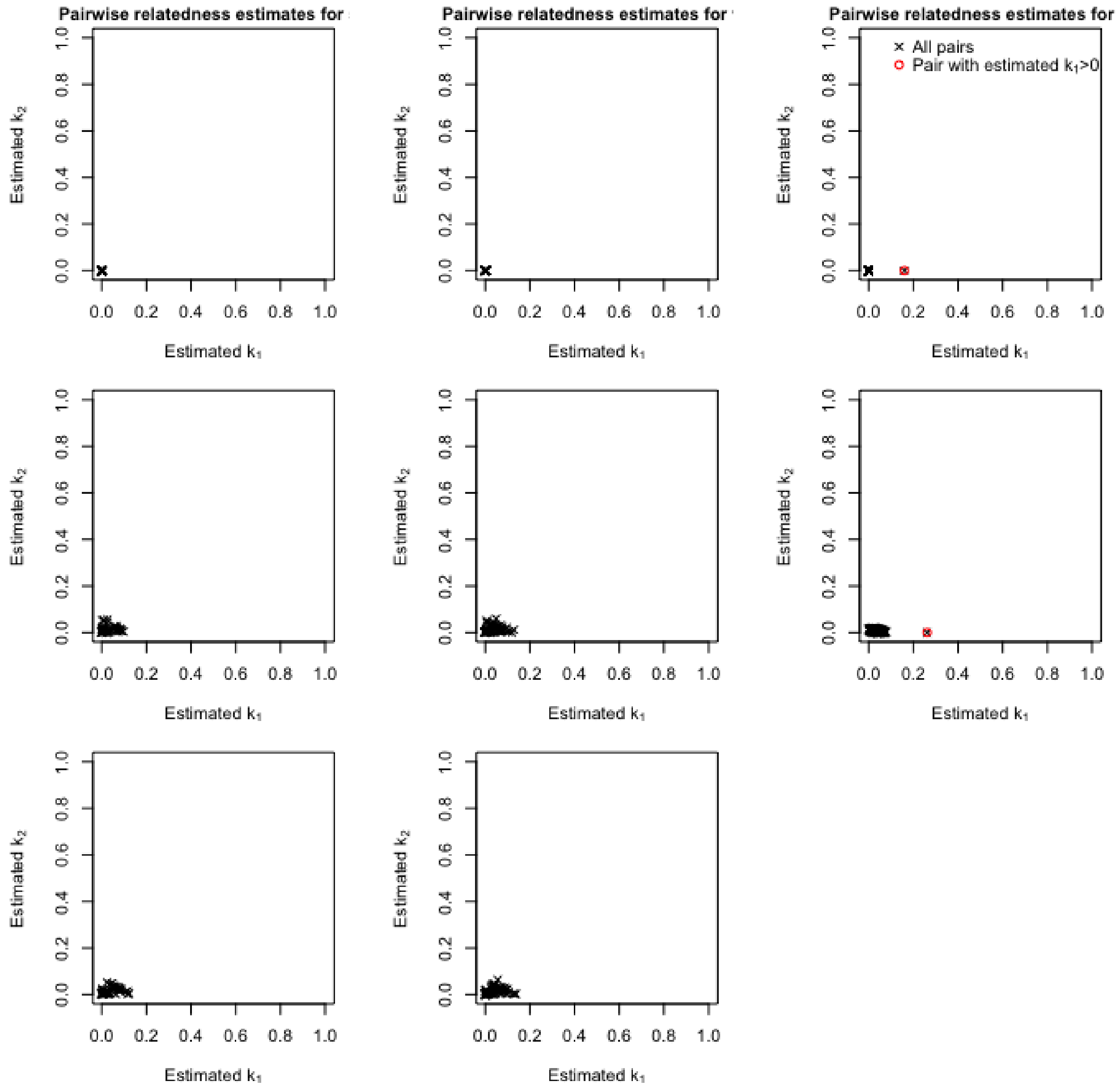

Figure S2. Relatedness estimates. Related to Figure 2. Plots of estimates of the relatedness coefficients k_1 and k_2 for all pair of individuals within each of the three datasets, sk (the first column), wf (the second column) and tr (the third column) with different estimates of allele frequency, which is based on the dataset itself (the first row), CEU population (the second row) and the modern samples of tr (the third row), respectively. In each of the plots there is a cross for each pair and the corresponding location on the x axis indicates the estimates of k_1 and k_2 obtained using ngsRelate. The expected values for an unrelated pair of individuals are $E(k_1)= E(k_2)=0$ and the expected values for first cousins are $E(k_1)=0.25$ and $E(k_2)=0$. The only one pair with an estimated k_1 above 0.15 (marked with a red circle) is a pair of tr samples (trondheim13 and trondheim27), which was estimated to have a k_1 value of 0.155 with the allele frequency of tr samples and 0.26 with the allele frequency of CEU.

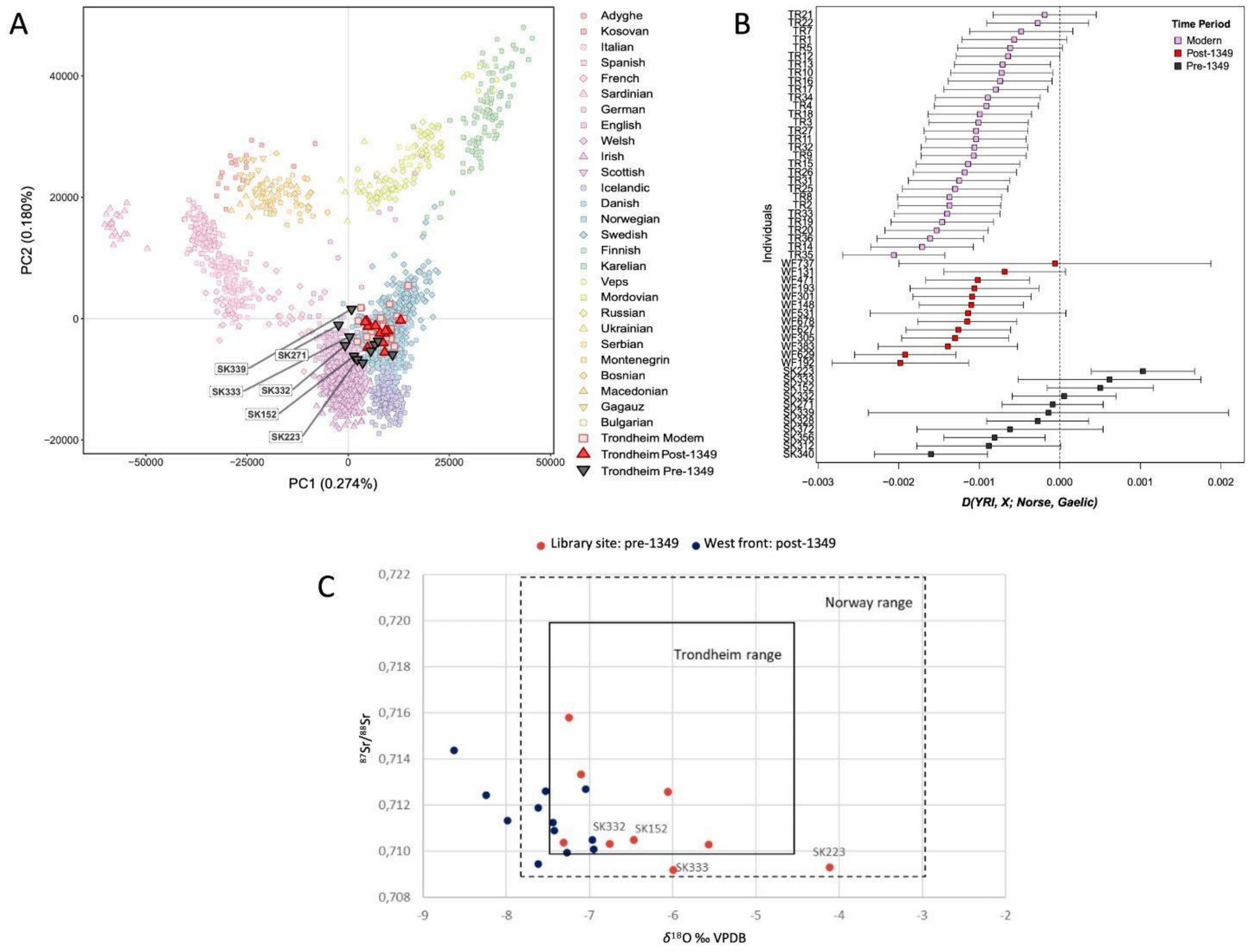

Figure S3. Genetic affinities and istotope analysis of origin of samples. Related to Figure 2. (A) Principal components analysis of all the ancient and modern Trondheim samples projected on the principal components estimated using modern European reference populations. (B) The genetic affinity of ancient and modern Trondheim individuals to contemporary Norse and Gaelic populations assessed using D-statistics. The horizontal error bars span ±3 standard errors from the mean estimate of the D-statistic. The standard errors were computed using the block jackknife procedure. (C) Strontium and Oxygen isotope results of the pre-1349 (red) and post-1349 (blue) cohorts. Pre-1349 individuals that are identified based on genomic analyses to contain elevated levels of Gaelic ancestry are labelled.

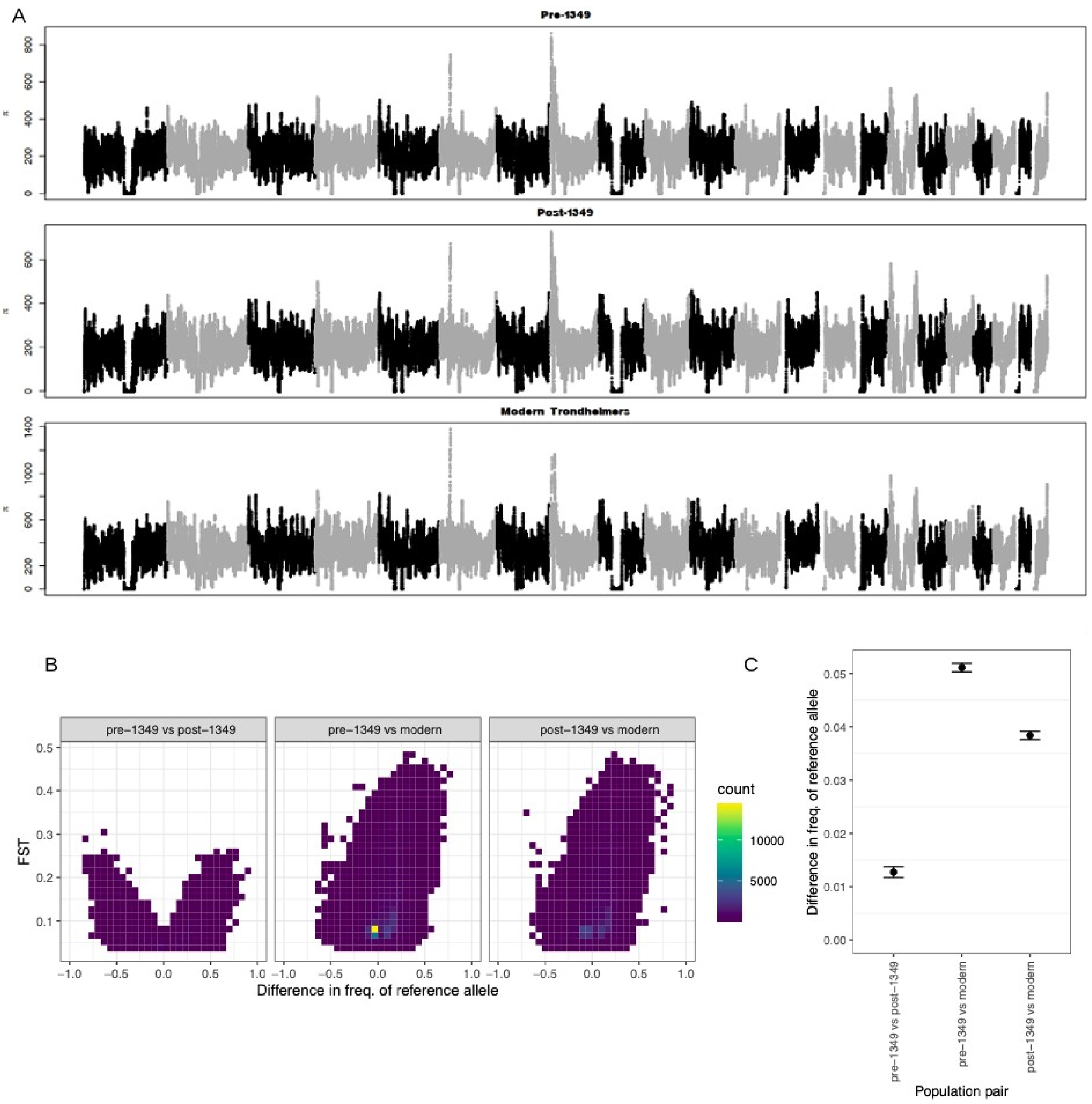

Figure S4: Nucleotide diversity and reference bias. Related to Figure 3. (A) Genome wide plot of the nucleotide diversity computed as pairwise differences (π) for the three populations. (B) The relationship between F_{ST} and Δf_{ref} across chromosome 1 when comparing the different Trondheim cohorts, left panel: pre- vs post-1349, middle panel: pre-1349 vs modern, and right panel: post-1349 vs modern. (C) The mean difference in allele frequency of the reference allele for the three cohort comparisons.

Table S1. Details of ancient samples. Related to Figure 1. Library site main phase date estimates are from [S1].West Front main phase date estimates are from [S2].

Skeleton	Site	Dating numbers from D-AMS (DirectAMS) OxA (Oxford Radiocarbon Accelerator Unit) TRa (NTNU Radiocarbon Accelator Unit)	δ(13C) per mill	AMS date 1σ error	Date, cal CE ± 1σ error	Site Phases	Age (Cultural Estimate, Main Phase)
sk152	Library	D-AMS008220<br>OxA-32178<br>TRa-13452	-20.2<br>-17.55	1039±30<br>984 ± 26<br>1055 ± 22	996 ± 30	B, 7, West	1225-1275
sk223	Library	OxA-33210	-17.43	1050 ± 28	988 ± 30	B, 6/7, Middle	1175-1275
sk271	Library	OxA-33212	-19.88	951 ± 28	1092 ± 40	B, 6/7, Middle	1175-1275
sk312	Library	Not dated			#N/A	A, 4/5, South	1100-1175
sk328	Library	Not dated			#N/A	B, 6, West	1175-1225
sk332	Library	Not dated			#N/A	B, 6, West	1175-1225
sk333	Library	OxA-33213	-17.42	1020 ± 29	1013 ± 32	A, 4/5, South	1100-1175
sk339	Library	Not dated			#N/A	A, 4/5, South	1100-1175
sk340	Library	OxA-33214	-18.79	1109 ± 37	930 ± 47	A, 4/5, South	1100-1175
sk356	Library	OxA-33215	-18.6	1005 ± 27	1031 ± 40	B, 6/7, Middle	1175-1275
sk372	Library	Not dated			#N/A	B, 6/7, South	1175-1275
wf131	West Front	OxA-33221	-20.76	189±24	1786 ± 91	22: The second level	1585-1897
wf148	West Front	OxA-33222	-20.1	119±24	1816 ± 78	23: The third level	1585-1897
wf192	West Front	Not dated			#N/A	22: The second level	1585-1897
wf193	West Front	OxA-33223	-18.13	219±24	1754 ± 92	22: The second level	1585-1897
wf301	West Front	D-AMS 008219	-22.5	148±27	1801 ± 84	26: The final use (1897)	1585-1897
wf305	West Front	OxA-33224	-19.88	127±24	1812 ± 80	22: The second level	1585-1897
wf383	West Front	OxA-33225	-18.07	190±26	1785 ± 91	23: The third level	1585-1897

wf471	West Front	OxA-33226	-19.32	152±25	1797 ± 84	22: The second level	1585-1897
wf531	West Front	OxA-32287	-18.89	142 ± 25	1804 ± 83	23: The third level	1585-1897
wf627	West Front	OxA-33227	-17.69	151±24	1798 ± 84	25: The re-interments	1585-1897
wf629	West Front	OxA-33228	-20.15	276±26	1599 ± 61	22: The second level	1585-1897
wf678	West Front	OxA-33229	-20.49	218±25	1756 ± 93	22: The second level	1585-1897
wf737	West Front	OxA-33230	-21.21	407±26	1485 ± 50	21: The earliest churchyard (1585)	1585-1897

Table S2: Data summary statistics. Related to Figure 2. Coverage on autosomes and sex chromosomes, including estimated mitochrondrial haplogroups, and Y chromosome haplotypes.

**Sample**	**Autosomal coverage (q30) [X]**	**X coverage (q30) [X]**	**Y coverage (q30) [X**	**Genetic sex - using R_y**	**Mitochondrial haplogroup**	**Y haplotype ISOGG 2018**
sk152	2.414	2.28	0.01	XX	H4a	
sk223	3.345	3.23	0.01	XX	J1b1a1a	
sk271	8.111	7.82	0.01	XX	H1bb	
sk312	0.466	0.47	0.01	XX	U5a2d1	
sk328	6.556	2.99	0.83	XY	H1a3a	R1b1a1a2a1a1c2b1b4e1a2c1
sk332	5.487	5.31	0.01	XX	R1a1a	
sk333	0.183	0.18	0.00	XX	J1b1a1a	
sk339	0.073	0.08	0.00	XX	H1o	
sk340	1.092	1.05	0.01	XX	T2e1	
sk356	7.145	7.05	0.03	XX	H10a1	
sk372	0.175	0.11	0.06	XY	U6a3	R1b1a1a2
tr1	35.822	14.31	14.27	XY	U5b1b1a3	R1b1a1a2a2
tr10	35.585	27.43	3.6	XX	H5	
tr11	30.866	13.37	13.05	XY	K1d	R1b1a1a2a1a1c2b2a1b1a1a2b2
tr12	33.333	14.53	15.34	XY	J1c7	J2a1b
tr13	9.512	14.21	11.62	XY	H1e2a	R1b1a1a2a1a1c2b1b4e1a2c1
tr14	38.215	16.14	15.15	XY	V	R1a1a1b1a3a2e4b
tr15	34.852	14.75	14.53	XY	H5a1g1a	I1a2a1a1d1a

tr16	36.204	30.96	3.75	XX	K1b2a2a		
tr17	38.834	17.18	15.92	XY	T2b2b	I1a1b1a4a2f1a1a1	
tr18	40.872	17.57	17.2	XY	J1c3(A189G)	I1a1b1a4a2	
tr19	37.324	30.34	3.86	XX	U5b1c1a		
tr2	33.748	25.99	3.66	XX	H13a1a		
tr20	36.611	15.35	14.93	XY	T2b1	R1a1a1b1a3a2a1	
tr21	33.233	14.28	13.79	XY	H5a1	R1b1a1a2a1a1b1a	
tr22	33.032	27.83	3.41	XX	H		
tr25	35.712	29.51	3.89	XX	J1c2a		
tr26	35.498	30.02	3.62	XX	J1c3		
tr27	30.651	26.01	3.39	XX	J1c2o		
tr3	37.873	14.83	14.86	XY	K1a(T195C)	I1a1b1a4a2f1a1a7	
tr31	36.353	29.82	3.59	XX	J1c2a		
tr32	36.365	30.71	4.05	XX	V14		
tr33	33.319	28.28	3.24	XX	H1a1b		
tr34	32.891	27.3	3.15	XX	U4a2		
tr35	36.003	31.11	3.81	XX	V		
tr36	32.555	28.28	3.57	XX	H1bn		
tr4	35.499	27.25	3.51	XX	H5a1k		
tr5	34.817	13.65	13.99	XY	H1b1	R1a1a1b1a3a1	
tr7	34.812	15.25	13.88	XY	H1(T152C)	E1b1b1b2a1a4d2c	

tr8	38.640	15.35	15.03	XY	V	R1b1a1a2a1a1b1b	
tr9	34.172	13.66	12.51	XY	V	R1b1a1a2a1a1g	
wf131	0.76	0.54	0.40	XY	T2b9	I1a	
wf148	8.054	3.87	2.23	XY	H1c3b	R1a1a1b1a3a2e1	
wf192	0.413	0.22	0.14	XY	Z1a1a	I1a1b1g	
wf193	0.454	0.22	0.12	XY	H	R1b1a1a	
wf301	0.6	0.42	0.27	XY	U5a1a1h	I1a	
wf305	3.693	1.15	0.71	XY	U5a1b3a	R1a1a1b1a3a3b1	
wf383	0.547	0.55	0.01	XX	U5a2a1(T152C)		
wf471	3.403	2.87	0.01	XX	U5b2a1a2		
wf531	0.196	0.20	0.08	N/A	H1n3	R1b1a1a2a1a	
wf627	3.634	3.13	0.01	XX	G2a(T152C)		
wf629	7.295	3.45	0.78	XY	U5a2c3a	N1a1a1a1a1a1a5	
wf678	6.171	5.75	0.01	XX	J1b1a1a		
wf737	0.062	0.06	0.01	XY	H28a		

Table S3. List of genes under peaks of Fst between pre-1349 and post-1349 samples. Related to Figure 3. The peaks were limited to 500 kb windows in the top 99.9% of all Fst windows across the genome. The genes were obtained by intersecting these regions with the genes from the ensembl 104 annotation. Start and end positions refer to coordinates in human genome assembly version GRCh38.

Chromosome	Start	End	Size	Genes
Chr 1	20640000	21480000	840000	PINK1, PINK1-AS, DDOST, KIF17, SH2D5, HP1BP3, EIF4G3, RNU7-200P, MIR1256, RPS15AP6, HSPE1P27, ECE1, ECE1-AS1, PPP1R11P1, PDE4DIPP10, NBPF2P, HS6ST1P1, CROCCP5, NBPF3, PFN1P10
Chr 1	25160000	25800000	640000	SYF2, RSRP1, RHD, SDHDP6, TMEM50A, RHCE, MACO1, LDLRAP1, MAN1C1
Chr 2	86860000	87360000	500000	CD8B, ANAPC1P1, RGPD1, WBP1P1, NDUFB4P5, PLGLB1, ANAPC1P2, ANAPC1P2, ANAPC1P3, MIR4771-1, CENPNP1, LINC01955, DBF4P3, IGKV3OR2-268
Chr 2	107220000	107940000	720000	LINC01789, LINC01885, LINC01886, GACAT1, RGPD4-AS1, RGPD4, RPL22P8, SRSF3P5
Chr 3	84000000	84800000	800000	LINC00971
Chr 3	87900000	88820000	920000	HTR1F, RNU6ATAC6P, CGGBP1, ZNF654, CBX5P1, C3orf38, ABCF2P1, CSNKA2IP, NDUFA5P5
Chr 3	93220000	93720000	500000	--
Chr 9	4800000	5500000	700000	RCL1, KLF4P1, MIR101-2, HNRNPA1P41, JAK2, CSNK1G2P1, PDSS1P1, MTND6P5, MTND1P11, MTCO1P11, MTCO2P11, MTATP6P11, MTCO3P11, MTND4P14, MTND5P14, TCF3P1, IGHEP2, INSL6, INSL4, RLN2, HMGN2P31, RLN1, PLGRKT, RNF152P1, CD274
Chr 15	48740000	49760000	1020000	CEP152, SHC4, EID1, KRT8P24, SECISBP2L, RN7SL577P, COPS2, GALK2, NDUFAF4P1, MIR4716, RN7SL307P, FAM227B, FGF7, DTWD1
Chr 20	34920000	35820000	900000	ACSS2, GSS, MYH7B, MIR499A, MIR499B, TRPC4AP, RNU6-407P, EDEM2, PROCR, RNA5SP483, MMP24OS, MT1P3, MMP24, EIF6, FAM83C-AS1, FAM83C, UQCC1, GDF5-AS1, GDF5, MIR1289-1, CEP250, CEP250-AS1, C20orf173, ERGIC3, RPL36P4, FER1L4, RPL37P1, SPAG4, CPNE1, RN7SKP271, RNU6-759P, RBM12, NFS1, ROMO1, RBM39, RPF2P1, U6, PHF20,

Supplemental References

S1. Anderson, T., and Göthberg, H. (1986). Olavskirkens kirkegård: humanosteologisk analyse og faseinndeling (Riksantikvaren, Utgravningskontoret for Trondheim).

S2. Reed, I., Kockum, J., Hughes, K., and Sandvik, P.U. (1998). Excavations outside the west front of Nidaros Cathedral in Trondheim. NIKU Oppdragsmelding *55*.
